# Renoprotective Effects of a Herbal Formulation Against Lipopolysaccharide-Induced Acute Kidney Injury Through Anti-Inflammatory and Antioxidant Activities

**DOI:** 10.3390/life16091442

**Published:** 2026-08-30

**Authors:** Chen-Xuan Du, Cheng-Wei Huang, Tsung-Han Lee, Zhu-Yin Wang, Hui-Chun Ku

**Affiliations:** Department of Life Science, Fu Jen Catholic University, New Taipei City 242, Taiwan

**Keywords:** NRICM101, AKI, NF-κB, NLRP3, TLR4, antioxidant, anti-inflammatory

## Abstract

Sepsis-associated acute kidney injury (AKI) is a severe inflammatory complication with limited therapeutic options. This study investigated the protective effects of Taiwan Chingguan Yihau (NRICM101), a herbal formulation, against lipopolysaccharide (LPS)-induced AKI. Male C57BL/6 mice were injected with LPS to induce AKI in vivo, followed by therapeutic administration of NRICM101. NRK-52E renal epithelial cells were stimulated with LPS to induce inflammatory responses and oxidative stress in vitro. Key bioactive compounds were identified via HPLC and analyzed using molecular docking. NRICM101 significantly alleviated renal dysfunction, as indicated by blood urea nitrogen (BUN) and serum creatinine (Scr) levels, and reduced histopathological damage in mice. It effectively suppressed the production of pro-inflammatory cytokines, attenuated intracellular and mitochondrial ROS, and preserved mitochondrial membrane potential. Mechanistically, NRICM101 markedly downregulated the expression of phosphorylated p65 (p-p65), NOD-like receptor protein 3 (NLRP3), and cleaved caspase-1, indicating modulation of nuclear factor kappa-B (NF-κB)/NLRP3 inflammasome signaling associated with renal pyroptosis. Molecular docking predicted potential interactions of NRICM101-derived bioactive constituents with the toll-like receptor 4 (TLR4)/MD-2 complex. Collectively, our findings suggest that NRICM101 ameliorates LPS-induced acute renal injury through anti-inflammatory and antioxidant effects associated with modulation of NF-κB/NLRP3-related signaling.

## 1. Introduction

Sepsis is a medical condition characterized by organ failure caused by an abnormal immune response to an infection [1]. The kidneys are particularly vulnerable, with approximately one-third of sepsis patients experiencing acute kidney injury (AKI) [2], referred to as sepsis-associated AKI [1]. Lipopolysaccharide (LPS), a molecule synthesized by Gram-negative bacteria, is a critical factor capable of inducing AKI [3]. LPS binding to toll-like receptor-4 (TLR4) activates nuclear factor kappa-B (NF-κB) through phosphorylation of the p65 subunit, driving the transcription of inflammatory genes [4]. This triggers cytokine secretion, including tumor necrosis factor-alpha (TNF-α) and interleukins (ILs), which recruit neutrophils and macrophages to the kidneys, increasing reactive oxygen species (ROS) production [5]. Simultaneously, inducible nitric oxide synthase (iNOS)-derived nitric oxide (NO) interacts with ROS to generate peroxynitrite, further intensifying oxidative stress [5]. This process results in the diminished efficacy of antioxidant scavenging enzymes [5]. Collectively, this inflammatory cascade induces mitochondrial dysfunction, exacerbating renal impairment [5]. AKI is characterized by a rapid decline in renal function, leading to electrolyte and fluid imbalances, nitrogenous waste accumulation, and high morbidity and mortality rates [2]. The diagnosis of AKI is established through the identification of elevated serum creatinine (Scr) and blood urea nitrogen (BUN) concentrations [2].

Pyroptosis, known as inflammatory cell necrosis, is a form of programmed cell death characterized by chromatin clumping, cell swelling, and the release of inflammatory cytokines and cellular contents [6]. NOD-like receptor protein-3 (NLRP3) plays a crucial role in pyroptosis. The activation of the NLRP3 inflammasome occurs in two steps [6]. The first step involves the upregulation of NF-kB signaling, which enhances the transcription of pro-inflammatory components such as NLRP3, pro-caspase-1, and cytokines such as pro-IL-1β and pro-IL-18 [7,8]. The second step is triggered by signals from ROS, mitochondrial dysfunction, and lysosomal breakdown, which promote the activation of the NLRP3 inflammasome [7,8]. Once activated, caspase-1 cleaves pro-IL-1β and pro-IL-18 into their mature forms, IL-1β and IL-18, respectively, while also cleaving gasdermin D (GSDMD) [6]. The cleaved GSDMD subsequently forms pores in the cell membrane, facilitating cytokine release and potassium efflux and ultimately leading to cell swelling and lysis [6]. Many studies indicate that pyroptosis significantly contributes to the injury of renal tubular epithelial cells in AKI, suggesting that inhibiting pyroptosis may be an effective treatment approach [9].

Taiwan Chingguan Yihau (NRICM101) is a ten-herb formulation [10] containing Scutellaria Root, Heartleaf Houttuynia, Mulberry Leaf, Saposhnikovia Root, Mongolian Snakegourd Fruit, Indigowoad Root, Liquorice Root, Magnolia Bark, Peppermint, and Fineleaf Nepeta [11]. Accumulating evidence has demonstrated that NRICM101 exerts immunomodulatory [12,13], anti-inflammatory [14], antioxidant [13], and neuroprotective activities [15]. Since excessive inflammation and oxidative stress are central mechanisms underlying AKI, we hypothesized that NRICM101 may confer renoprotective effects by attenuating these pathological processes. Nevertheless, the therapeutic potential and molecular mechanisms of NRICM101 in AKI have not yet been elucidated. In addition, no effective treatments have been proven to ameliorate AKI, except for renal support through dialysis [16]. Novel therapeutic agents targeting AKI need to be developed. Therefore, this study aimed to evaluate the renoprotective effects of NRICM101 in a mouse model of AKI and investigate the associated molecular signaling pathways.

## 2. Materials and Methods

### 2.1. Preparation of NRICM101 Extract

NRICM101, supplied by Chuan Feng Tang Pharmaceutical Co., Ltd. (Taipei, Taiwan), was confirmed to be endotoxin-free. The formulation comprised 10 herbs in defined amounts: Scutellaria Root (*Scutellaria baicalensis*), 18.75 g; Heartleaf Houttuynia (*Houttuynia cordata*), 18.75 g; Mulberry Leaf (*Morus alba*), 11.25 g; Saposhnikovia Root (*Saposhnikovia divaricata*), 7.50 g; Mongolian Snakegourd Fruit (*Trichosanthes kirilowii*), 18.75 g; Indigowoad Root (*Isatis indigotica*), 18.75 g; baked Liquorice Root (*Glycyrrhiza glabra*), 7.50 g; Magnolia Bark (*Magnolia officinalis*), 11.25 g; Peppermint Herb (*Mentha haplocalyx*), 11.25 g; and Fineleaf Nepeta (*Nepeta tenuifolia*), 11.25 g [11]. For the preparation of the standard daily dose for human use, a complete set of herbs was decocted in 1 L of water and simmered until the final volume was reduced to 300 mL [11]. The resulting decoction was filtered and centrifuged at 5000 rpm for 10 min. The aqueous extract was concentrated under reduced pressure using a rotary evaporator (EYELA N-1200, Tokyo Rikakikai Co., Ltd., Tokyo, Japan) to achieve a total soluble solid content of approximately 10–15% (*w*/*v*). The concentrated extract was subsequently freeze-dried using a FreeZone 18 L freeze dryer (Labconco Inc., Kansas City, MO, USA) at a reduced pressure of 65 Pa for 24 h. The resulting yield was 15.9 ± 0.8 g of crude freeze-dried extract per 300 mL of decoction. The total flavonoid content of the NRICM101 extract was 28.75 μg quercetin equivalents (QE)/mg of extract.

### 2.2. Experimental Model of LPS-Induced AKI

All animal procedures followed the guidelines outlined in the Guide for the Care and Use of Laboratory Animals issued by the U.S. National Institutes of Health (NIH Publication No. 85–23, revised 1996). The experimental protocol was reviewed and approved by the Institutional Animal Care and Use Committee of Fu Jen Catholic University, Taiwan (IACUC approval no. A11236). A total of 33 eight-week-old male C57BL/6 mice (BioLASCO Taiwan Co., Ltd., Taipei, Taiwan), weighing 22.0–23.5 g, were used in this study. The mice were randomly allocated to five groups: control (*n* = 6), LPS (*n* = 7), LPS + NRICM101 (1 g/kg; *n* = 7), LPS + NRICM101 (1.5 g/kg; *n* = 7), and LPS + NRICM101 (3 g/kg; *n* = 6). AKI was induced by a single intraperitoneal injection of LPS (10 mg/kg; Escherichia coli O111:B4, L3024, Sigma-Aldrich, St. Louis, MO, USA), as previously described [17]. Four hours after LPS injection, NRICM101 was administered by oral gavage at doses of 1, 1.5, or 3 g/kg, as previously described [14,18]. The control group received an equivalent volume of isotonic saline. One mouse in each of the LPS, LPS + NRICM101 (1 g/kg), and LPS + NRICM101 (1.5 g/kg) groups died following LPS administration; therefore, six surviving mice from each group were included in the final analyses (*n* = 6 per group). After 24 h of LPS exposure, the mice were euthanized using CO_2_, and blood and kidney tissues were collected for subsequent analyses.

### 2.3. Renal Histology

The kidneys were fixed in 4% paraformaldehyde, embedded in paraffin, and sectioned horizontally into 4-μm-thick sections using a rotary microtome. Hematoxylin and eosin (H&E) staining was performed to evaluate overall renal morphology and histopathological alterations, including tubular injury and inflammatory cell infiltration, whereas periodic acid–Schiff (PAS) staining was used to further assess tubular structural integrity, particularly the integrity of the tubular brush border. Histological sections were examined and imaged using an Olympus light microscope (Olympus, Tokyo, Japan). Histopathological assessment was performed by a single pathologist who was blinded to the experimental group allocation. Renal tubular injury was assessed semi-quantitatively based on the percentage of pathologically damaged areas within each field of view and graded on a scale of 0–4 as follows: 0, no detectable injury; 1, <25% damaged area; 2, 25–50% damaged area; 3, 50–75% damaged area; and 4, >75% damaged area [19]. Ten randomly selected cortical fields were evaluated for each animal, and the mean score of the 10 fields was calculated as the final tubular injury score for each mouse.

### 2.4. Determination of Renal Function

Blood samples were collected from the vena cava at the time of sacrifice into heparin-rinsed collection tubes without additional anticoagulant supplementation. After centrifugation, the plasma was collected and stored at −80 °C until analysis. BUN and Scr levels were measured using a cobas 6000 automated analyzer equipped with the cobas c 501 module (Roche Diagnostics, Mannheim, Germany) and the corresponding Roche cobas c pack reagents according to the manufacturer’s instructions.

### 2.5. RNA Extraction and Reverse Transcription Quantitative Polymerase Chain Reaction (RT-qPCR)

Total RNA was isolated from kidney tissues and NRK52E cells using TRIzol reagent (Thermo Fisher Scientific, Waltham, MA, USA) according to the manufacturer’s instructions. Total RNA was reverse transcribed using the Maxima First Strand cDNA Synthesis Kit (Thermo Fisher Scientific, Waltham, MA, USA), and quantitative real-time PCR was performed using SYBR Green (Bio-Rad Laboratories, Hercules, CA, USA)**.** The sequences of the forward and reverse primers used for mouse kidney tissues and rat NRK52E cells are provided in Supplementary Tables S1 and S2, respectively. The expression level of each transcript was normalized to *HPRT* and expressed relative to the mean expression level of the control samples. All primer pairs demonstrated amplification efficiencies of 95–103%, with R^2^ values > 0.99. HPRT Cq values remained stable across experimental groups (SD = 0.32 cycles), with no significant differences among groups (*p* > 0.05). Primer specificity was confirmed by NCBI Primer-BLAST and melting-curve analysis.

### 2.6. Measurement of Antioxidant Enzyme Activity

Kidney tissues were homogenized in ice-cold phosphate-buffered saline at a 1:10 tissue-to-buffer ratio (*w*/*v*), followed by centrifugation at 12,000× *g* for 15 min at 4 °C. The resulting supernatant, representing the post-mitochondrial fraction, was collected for the assessment of antioxidant enzyme activities. Total protein concentration in the supernatant was determined using a BCA protein assay kit with bovine serum albumin as the standard, and antioxidant enzyme activities were normalized to total protein content. Superoxide dismutase (SOD) and glutathione peroxidase (GPx) activities were measured with RANSOD and RANSEL kits (Randox Laboratories Ltd., Crumlin, UK), respectively, following the protocols provided by the manufacturer. Catalase activity was assessed using the Catalase Assay Kit (Item No. 707002, Cayman Chemical, Ann Arbor, MI, USA). In this assay, catalase reacts with methanol in the presence of hydrogen peroxide through its peroxidatic activity, generating formaldehyde that is subsequently quantified colorimetrically with Purpald as the chromogen.

### 2.7. Measurement of Nitrate Levels

Nitrate, a stable metabolite of nitric oxide (NO), was used as an index of NO production. Before analysis, serum samples were subjected to ultrafiltration through Amicon Ultra centrifugal filter units with a 10-kDa molecular weight cutoff to remove proteins. Nitrate concentrations were then measured using a Nitric Oxide Colorimetric Assay Kit (Catalog No. K262-200, BioVision, Milpitas, CA, USA) following the manufacturer’s protocol. Nitrate was first enzymatically reduced to nitrite by nitrate reductase. The generated nitrite subsequently reacted with Griess reagents to form a colored azo product, which was quantified by measuring its absorbance.

### 2.8. Measurement of Total Antioxidant Capacity

Serum total antioxidant capacity was determined using a Total Antioxidant Capacity Assay Kit (ab65329, Abcam, Cambridge, UK) according to the manufacturer’s protocol. The assay is based on the reduction of Cu^2+^ to Cu^+^ by antioxidants in the sample. The resulting colorimetric signal was measured at 570 nm using a microplate reader, and total antioxidant capacity was expressed as mM Trolox equivalents.

### 2.9. Immunohistochemical (IHC) Staining

IHC staining was carried out as previously described [20]. Primary antibodies (p-p65, 1:200; NLRP3, 1:100; cleaved caspase-1, 1:100; Abcam) were applied overnight at 4 °C. Detection was performed using a horseradish peroxidase-conjugated secondary antibody for 30 min at room temperature, followed by chromogenic substrate development and hematoxylin counterstaining. For quantitative analysis of IHC staining, 10 images of the renal cortex were analyzed per animal. The immunopositive staining was quantified using ImageJ software (version 1.54g).

### 2.10. Cell Culture

NRK-52E cells, which are rat tubular epithelial cells, were obtained from the American Type Culture Collection. The cells were cultured in Dulbecco’s Modified Eagle Medium supplemented with 5% bovine calf serum (Gibco, Scotland, UK) and antibiotics (100 μg/mL penicillin and 100 μg/mL streptomycin; Amresco, Solon, OH, USA), and were incubated at 37 °C in a 5% CO_2_/95% air atmosphere. The cells were treated with LPS (Sigma-Aldrich, St. Louis, MO, USA; concentrations of 0.1, 0.3, 0.5, and 1 μg/mL) and adenosine triphosphate (ATP) (Sigma-Aldrich, St. Louis, MO, USA; 5 mM) for 24 h. In the treated groups, NRICM101 (0.125, 0.25, and 0.5 mg/mL) was administered 30 min prior to LPS exposure. To investigate the signaling pathway, BMS-986299, an NLRP3 agonist, was incubated for 1 h before the administration of LPS.

### 2.11. Cell Viability Assay

Cell viability was assessed using a 3-(4,5-Dimethylthiazol-2-yl)-2,5-diphenyltetrazolium bromide (MTT) assay (0.5 mg/mL); formazan crystals were solubilized in DMSO and quantified at 570 nm using a microplate reader.

### 2.12. Immunofluorescent Staining

P-p65 and NLRP3 expression in cells was examined using immunofluorescence. The cells were incubated overnight at 4 °C with p-p65 and NLRP3 antibodies diluted 1:50 (Abcam, Waltham, MA, USA). The immunofluorescence staining procedure was performed as previously described [20].

### 2.13. Measurement of Lactate Dehydrogenase (LDH) Release

Cell injury was assessed by measuring LDH release into the culture supernatant using the CytoTox 96^®^ Non-Radioactive Cytotoxicity Assay (Promega, Madison, WI, USA; Cat. No. G1780) according to the manufacturer’s instructions. After treatment, culture supernatants were collected and centrifuged to remove floating cells and debris. Spontaneous LDH release was determined from untreated cells, whereas maximum LDH release was determined from completely lysed cells. Cell-free supernatants were incubated with the substrate mixture for 30 min at room temperature, and absorbance was measured at 490 nm. LDH release was calculated as follows: LDH release (%) = [(OD_experimental − OD_spontaneous)/(OD_maximum − OD_spontaneous)] × 100.

### 2.14. Detection of Activated Caspase-1

Activated caspase-1 was detected using the FAM-VAD-FMK probe (Abcam, USA). Fluorescence inside the cells was observed through fluorescence microscopy, employing an excitation wavelength of 395 nm and an emission wavelength of 509 nm. The procedure was repeated in three independent experiments.

### 2.15. Detection of ROS Production and Mitochondrial Membrane Potential

Intracellular and mitochondrial ROS, along with mitochondrial membrane potential, were measured in NRK52E cells using dihydroethidium (DHE), MitoSOX, and tetramethylrhodamine methyl ester (TMRM) (Thermo Fisher Scientific). The fluorescence intensity was calculated by averaging the fluorescence intensity of multiple outlined cells using ImageQuant (Molecular Dynamics, Inc., Sunnyvale, CA, USA).

### 2.16. Fingerprint Analysis for NRICM101

The NRICM101 decoction was subjected to high-performance liquid chromatography (HPLC) analysis for the identification and quantification of selected constituents. The sample was filtered through a 0.45 μm syringe filter prior to HPLC analysis. The five compounds analyzed in the present study were selected based on previous phytochemical studies of NRICM101 [13,15]. Gradient elution was applied for the identification and quantification of the selected compounds. At 270 nm, oroxylin A-7-O-glucuronide and wogonin-7-O-glucuronide were detected at retention times of 40.39 and 42.53 min, respectively. Liquiritin and baicalin were detected at 280 nm, with retention times of 25.94 and 34.61 min, respectively, whereas rosmarinic acid was detected at 320 nm with a retention time of 29.44 min. For method validation, baicalin was selected as a representative chemical marker based on previous phytochemical characterization demonstrating its consistent quantification across 12 batches of NRICM101 decoction [13]. Baicalin showed good linearity over the concentration range of 0.015–2 mg/mL (R^2^ = 0.9998), with recovery rates ranging from 97.2% to 102.5%. The limit of detection for the analyzed compounds was 0.005 mg/mL, and the limit of quantification was defined at a signal-to-noise ratio of 10:1.

### 2.17. Molecular Docking

The crystal structure of the human TLR4/MD-2 complex was retrieved from the Protein Data Bank (PDB ID: 3FXI) and prepared using UCSF Chimera (v1.19) by removing non-essential heteroatoms, adding polar hydrogens, and performing energy minimization with the Amber force field. The 3D structures of the bioactive constituents of NRICM101 were obtained from the PubChem database and prepared using AutoDockTools (v1.5.7). Molecular docking was performed using AutoDock Vina (v1.2.7), targeting the hydrophobic LPS-binding cavity of MD-2. The grid box was centered at X = 29.000, Y = −7.000, and Z = 17.875, with dimensions of approximately 15.4 × 19.9 × 31.1 Å^3^, based on a previously reported docking protocol for human MD-2 (PDB ID: 3FXI) [21]. Docking validation was performed by re-docking the co-crystallized ligand into the MD-2 binding pocket. The redocked pose showed a root-mean-square deviation of 0.639 Å from the crystallographic pose. Following validation, the NRICM101 constituents were docked using the same parameters. The optimal docking poses were selected based on the lowest predicted binding energies (kcal/mol), with values below −7.0 kcal/mol considered indicative of favorable binding [22,23]. Ligand–protein interactions were visualized and analyzed using UCSF Chimera.

### 2.18. Statistical Analyses

All statistical analyses were conducted using GraphPad Prism 8.0 (GraphPad Software, San Diego, CA, USA), and results are expressed as the mean ± SEM. Data normality was evaluated using the Shapiro–Wilk test. Differences among multiple groups were analyzed by one-way ANOVA followed by Tukey’s multiple-comparison test. For the semi-quantitative tubular injury scores, differences among groups were analyzed using the Kruskal–Wallis test followed by Dunn’s multiple-comparison test. Statistical significance was defined as *p* < 0.05.

## 3. Results

### 3.1. NRICM101 Alleviates Pathological Damage in Mice with LPS-Induced AKI

**Figure 1:**
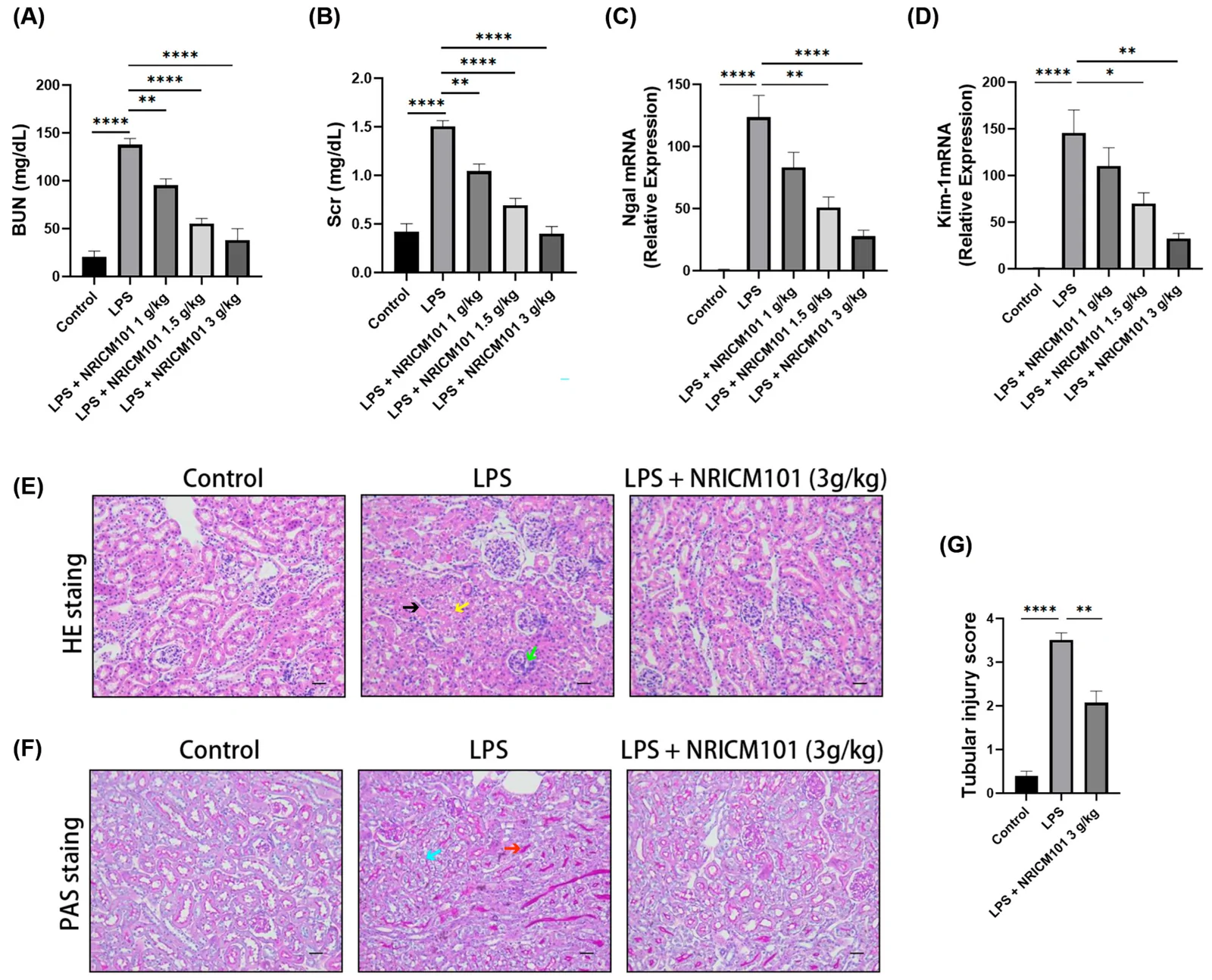
Effects of NRICM101 on LPS-induced AKI. (**A**) BUN and (**B**) Scr levels were measured. RT-qPCR analysis was performed to determine the mRNA expression levels of (**C**) NGAL and (**D**) KIM-1. Representative kidney sections stained with (**E**) H&E and (**F**) PAS. (**G**) Semi-quantitative tubular injury scores. Black arrows indicate tubular swelling; yellow arrows, loss of epithelial integrity; green arrows, inflammatory cell infiltration; blue arrows, disruption of the brush border; and red arrows, cellular debris. Scale bar = 50 μm. (*n* = 6). * *p* < 0.05, ** *p* < 0.01, and **** *p* < 0.0001.

We initially investigated the biological effects of NRICM101 on AKI in vivo using an LPS-induced mouse model. LPS significantly increased serum BUN (Figure 1A) and Scr (Figure 1B) levels, while NRICM101 dose-dependently attenuated these elevations, indicating improved renal function. To further evaluate kidney injury, the mRNA expression levels of NGAL (Figure 1C) and KIM-1 (Figure 1D) were measured by RT-qPCR. LPS markedly increased the mRNA expression levels of both NGAL and KIM-1, whereas NRICM101 significantly attenuated these elevations. Based on these results, a dose of 3 g/kg/day was selected for subsequent experiments.

Histopathological examination further demonstrated LPS-induced renal injury. H&E staining showed preserved renal tubular and glomerular morphology in the control group. In contrast, LPS exposure induced prominent acute tubular injury, predominantly affecting the proximal tubules, characterized by marked tubular epithelial swelling, loss of epithelial integrity, luminal accumulation of sloughed cellular debris, and interstitial inflammatory cell infiltration (Figure 1E). These histopathological alterations were markedly attenuated by NRICM101 treatment, with improved tubular epithelial integrity, reduced cellular swelling and inflammatory cell infiltration, and largely restored tubular lumens.

PAS staining was further performed to evaluate the integrity of the proximal tubular brush border. Kidneys from control mice exhibited a continuous PAS-positive apical brush border along the proximal tubular epithelium. In contrast, LPS exposure resulted in marked disruption and loss of the brush border, accompanied by intraluminal accumulation of PAS-positive casts and cellular debris and flattening of the tubular epithelium (Figure 1F). NRICM101 treatment substantially preserved tubular architecture, with reduced intraluminal casts and debris and partial restoration of the PAS-positive brush border.

Consistent with these morphological findings, semi-quantitative analysis demonstrated a significant increase in the tubular injury score in the LPS group compared with the control group, whereas NRICM101 treatment significantly reduced the LPS-induced tubular injury score (Figure 1G).

### 3.2. NRICM101 Reduces the Inflammatory Response in Mice with LPS-Induced AKI

**Figure 2:**
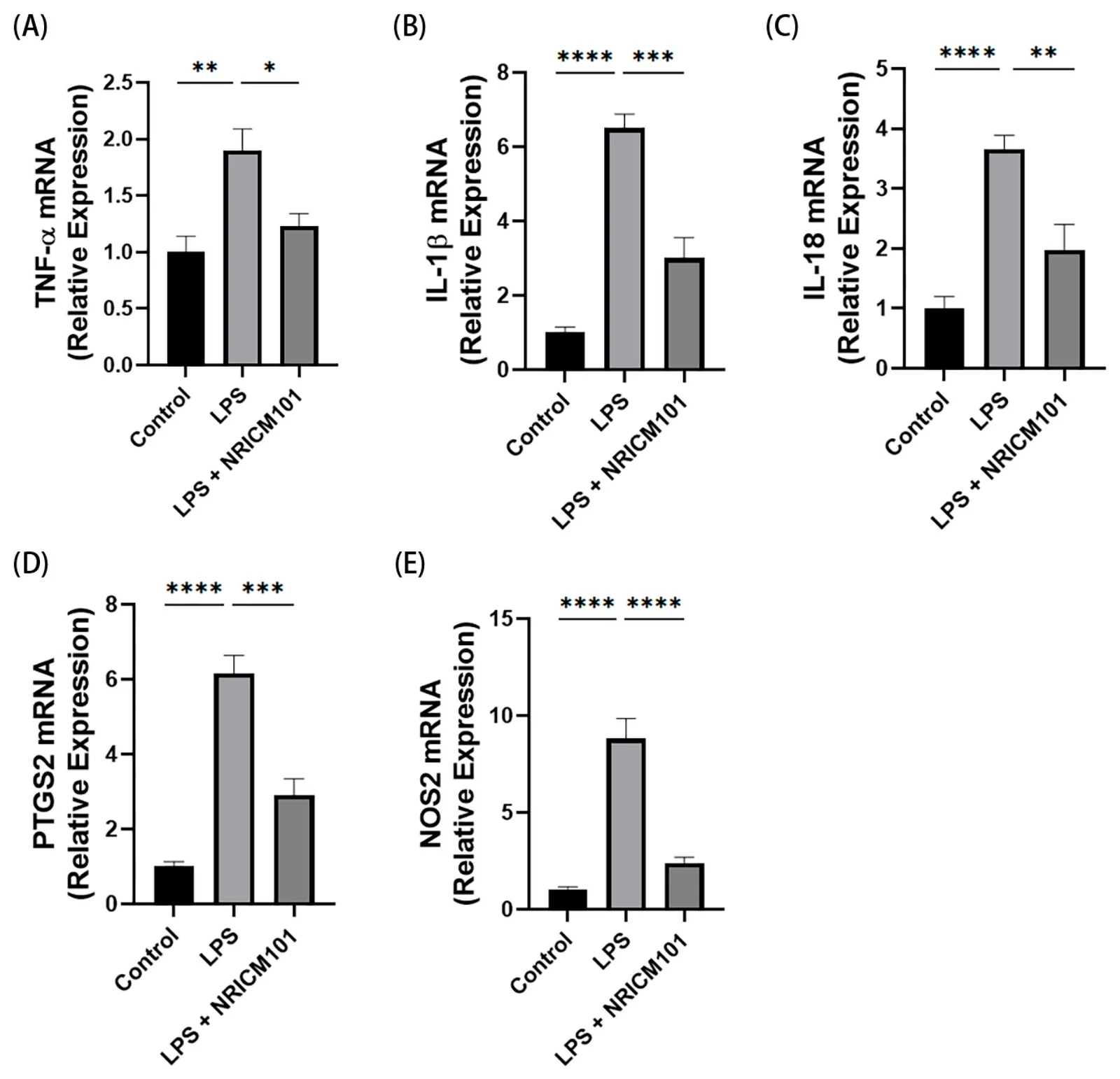
Effects of NRICM101 on LPS-induced inflammatory response in AKI. RT-qPCR analysis was performed to determine the mRNA expression levels of (**A**) TNF-α, (**B**) IL-1β, (**C**) IL-18, (**D**) PTGS2, (**E**) NOS2. (*n* = 6) * *p* < 0.05, ** *p* < 0.01, *** *p* < 0.001, **** *p* < 0.0001.

LPS triggers systemic cytokine storms and inflammatory mediator overactivation. RT-qPCR analysis demonstrated that LPS treatment significantly elevated the levels of cytokine genes, including TNF-α (Figure 2A), IL-1β (Figure 2B) and IL-18 (Figure 2C), in the kidneys compared to the control group. Notably, NRICM101 reduced the upregulation of these cytokine genes. In addition, the mRNA levels of PTGS2 (Figure 2D) and NOS2 (Figure 2E) were increased following LPS exposure, whereas NRICM101 treatment significantly reduced their expression levels.

### 3.3. NRICM101 Alleviates Oxidative Stress in Mice with LPS-Induced AKI

**Figure 3:**
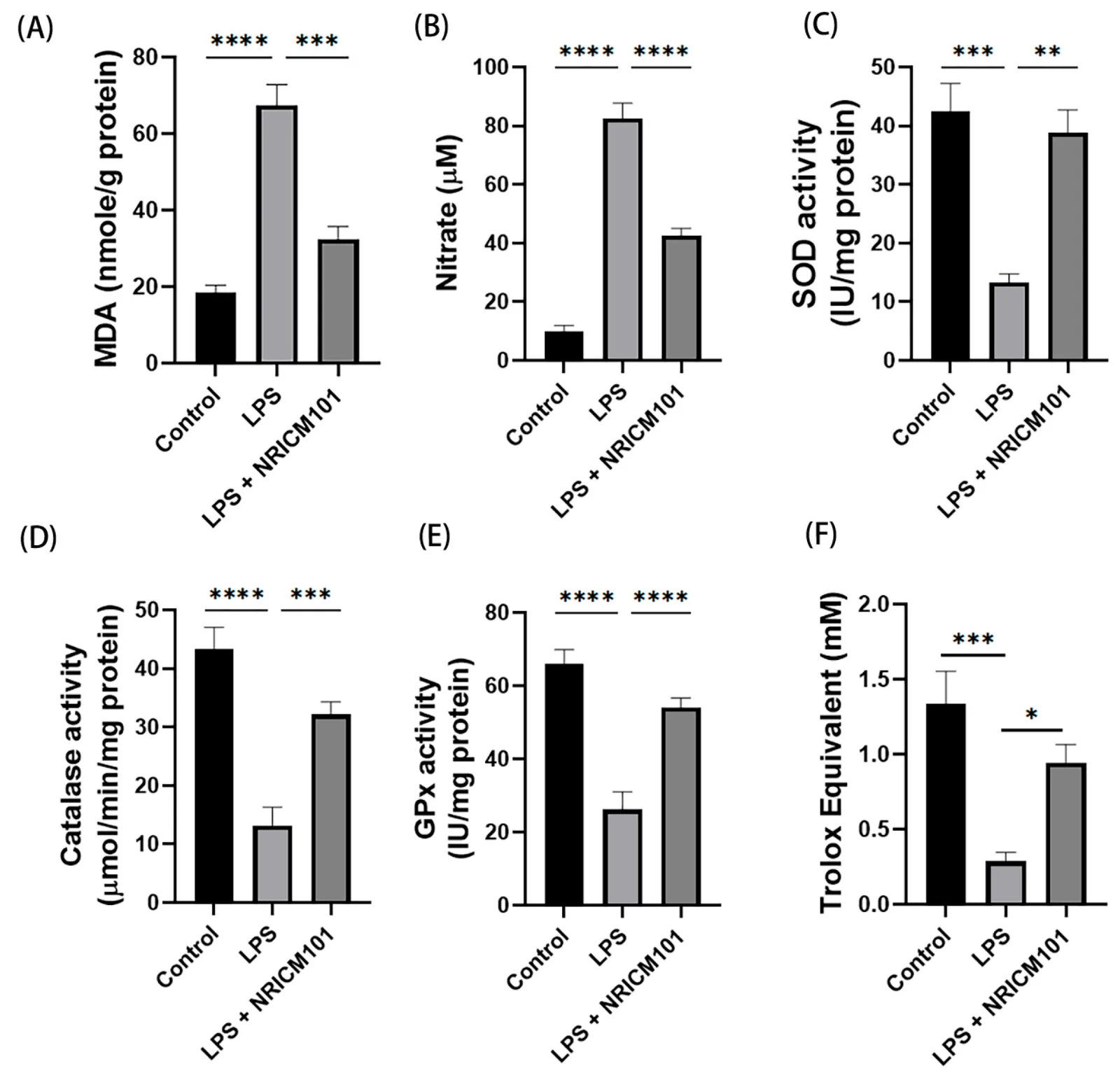
Effects of NRICM101 on LPS-induced oxidative stress in AKI. (**A**) Renal lipid peroxidation was determined by measuring malondialdehyde (MDA) levels. (**B**) Serum NO levels were determined by measuring nitrate levels. The activities of (**C**) SOD, (**D**) catalase, and (**E**) GPx in kidney tissues were measured. (**F**) Total antioxidant capacity was measured in serum. (*n* = 6) * *p* < 0.05, ** *p* < 0.01, *** *p* < 0.001, **** *p* < 0.0001.

LPS induced oxidative stress, as evidenced by increased lipid peroxidation (Figure 3A) and nitric oxide levels (Figure 3B) and decreased renal antioxidant enzyme activities, including SOD, catalase, and GPx (Figure 3C–E). In addition, LPS exposure significantly reduced serum total antioxidant capacity (Figure 3F). NRICM101 treatment significantly attenuated all of these LPS-induced alterations, supporting its antioxidant effects.

### 3.4. NRICM101 Suppresses Pyroptosis-Associated Signaling in Mice with LPS-Induced AKI

**Figure 4:**
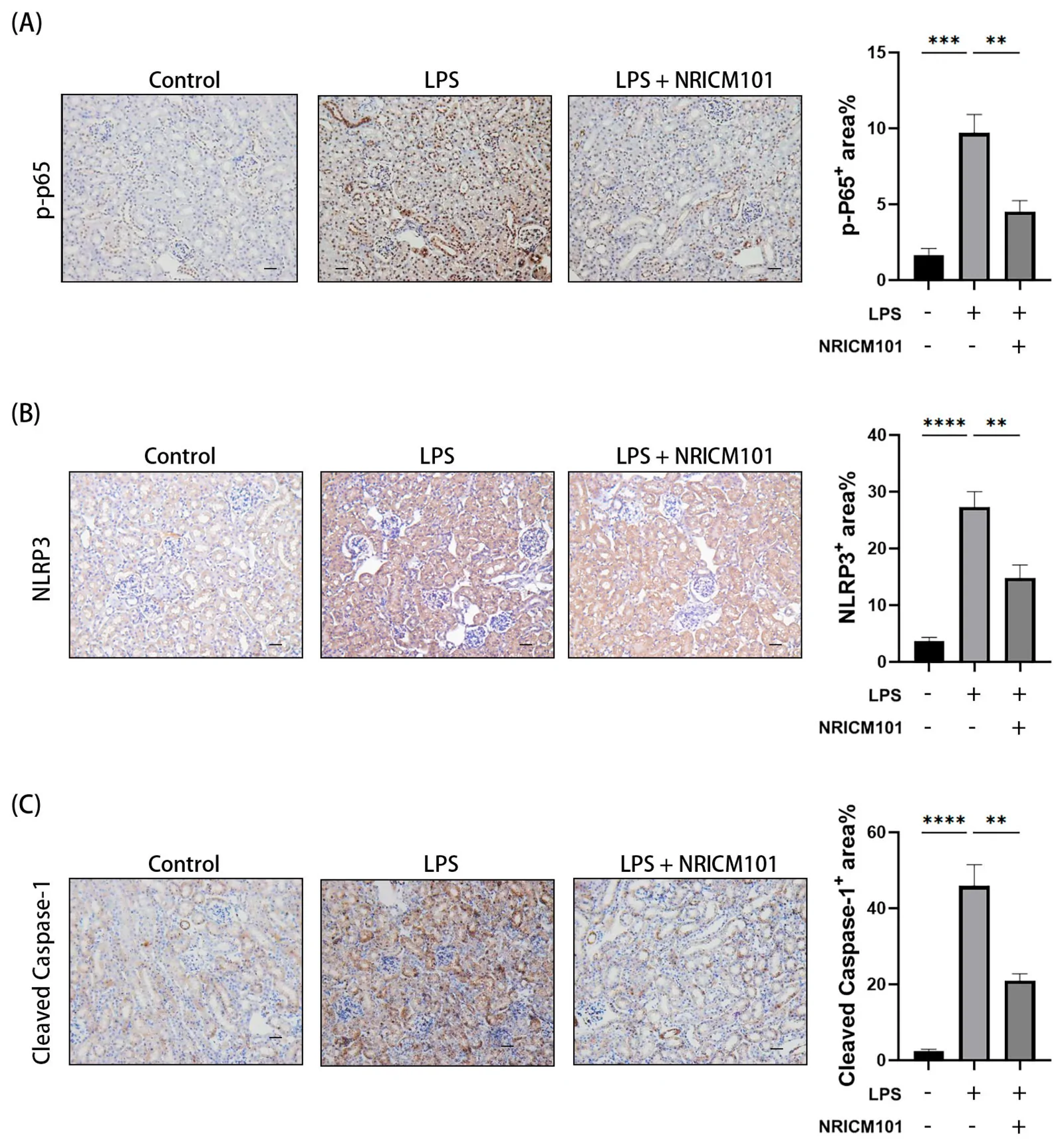
Effects of NRICM101 on LPS-induced pyroptosis-associated signaling in AKI. IHC analyses of (**A**) p-p65, (**B**) NLRP3, and (**C**) cleaved caspase-1 expression in kidney tissues. Scale bar = 50 μm (*n* = 6) ** *p* < 0.01, *** *p* < 0.001, **** *p* < 0.0001.

We then assessed the downstream signaling triggered by LPS, concentrating on the primary signaling pathway involving the NF-κB protein. Immunohistochemical (IHC) showed that LPS elevated the expression levels of p-p65 (Figure 4A) in the kidneys of mice compared to the control group. However, treatment with NRICM101 significantly decreased the LPS-induced p-p65 expression.

To determine whether NRICM101 influences pyroptosis related to AKI, we examined the expression of proteins linked to this process. LPS increased renal NLRP3 (Figure 4B) and cleaved caspase-1 (Figure 4C) expression, whereas NRICM101 significantly suppressed these elevations, indicating modulation of pyroptosis-associated signaling in AKI.

### 3.5. NRICM101 Protects NRK52E Cells Against LPS-Induced Cytotoxicity

**Figure 5:**
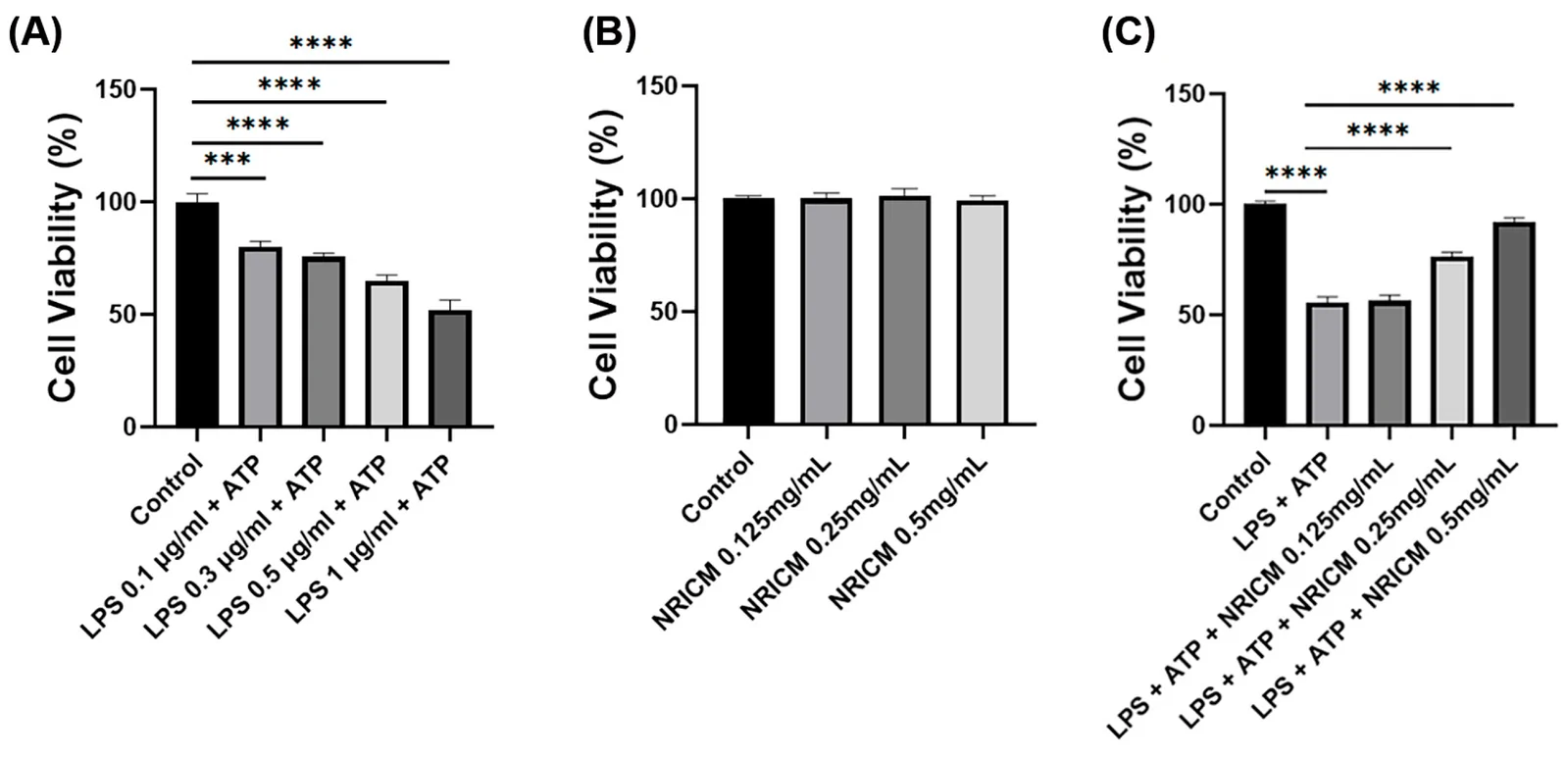
Effects of NRICM101 on cell viability in NRK52E cells. (**A**) Cell viability was assessed after treatment with LPS (0.1, 0.3, 0.5, and 1 μg/mL) combined with ATP (5 mM). (**B**) Cell viability was evaluated following treatment with NRICM101 alone at concentrations of 0.125, 0.25, and 0.5 mg/mL. (**C**) Cell viability was evaluated following treatment with LPS (1 μg/mL) and ATP (5 mM) in the presence of varying concentrations of NRICM101 (0.125, 0.25, and 0.5 mg/mL). (*n* = 4) *** *p* < 0.001, **** *p* < 0.0001.

To evaluate the protective effect of NRICM101 in renal epithelial cells, NRK52E cells were exposed to increasing concentrations of LPS (0.1–1 μg/mL) in combination with ATP (5 mM). Cell viability progressively decreased with increasing LPS concentrations (Figure 5A). Based on these findings, 1 μg/mL LPS combined with 5 mM ATP was used in subsequent experiments. Treatment with NRICM101 alone (0.125–0.5 mg/mL) did not significantly affect cell viability (Figure 5B). In LPS/ATP-stimulated cells, NRICM101 (0.125–0.5 mg/mL) significantly restored cell viability in a concentration-dependent manner, with 0.5 mg/mL showing the greatest protective effect (Figure 5C). Therefore, 0.5 mg/mL NRICM101 was selected for subsequent experiments.

### 3.6. NRICM101 Attenuates NLRP3/Caspase-1-Associated Signaling in NRK52E Cells

**Figure 6:**
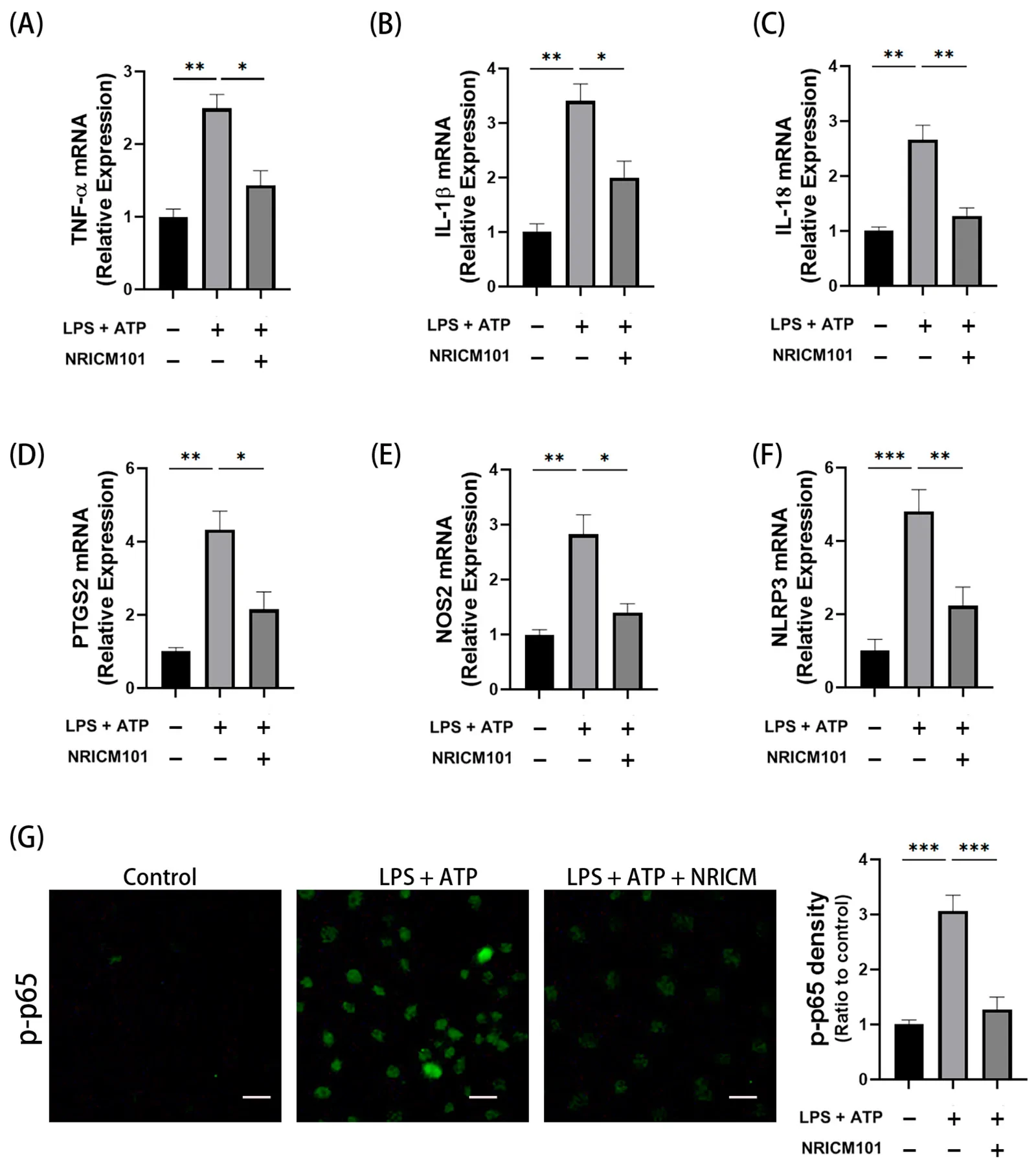
Effects of NRICM101 on LPS-induced inflammatory responses in NRK52E cells. Cells were treated with LPS (1 μg/mL) and ATP (5 mM) for 24 h, either in the presence or absence of NRICM101 (0.5 mg/mL). RT-qPCR analysis was performed to determine the mRNA expression levels of (**A**) TNF-α, (**B**) IL-1β, (**C**) IL-18, (**D**) PTGS2, (**E**) NOS2, and (**F**) NLRP3. (**G**) Representative immunofluorescence staining of p-p65 expression. Scale bar = 25 μm. (*n* = 4) * *p* < 0.05, ** *p* < 0.01, *** *p* < 0.001.

To further validate the anti-inflammatory properties of NRICM101, we assessed inflammatory mediators in cells stimulated with LPS. LPS increased the expression of pro-inflammatory mediators, such as TNF-α (Figure 6A), IL-1β (Figure 6B), IL-18 (Figure 6C), PTGS2 (Figure 6D), NOS2 (Figure 6E), NLRP3 (Figure 6F), and NF-κB activation (p-p65) (Figure 6G), whereas NRICM101 attenuated these changes, indicating modulation of NF-κB/NLRP3-associated signaling.

### 3.7. NRICM101 Attenuates NLRP3/Caspase-1-Associated Signaling in LPS-Treated NRK52E Cells

**Figure 7:**
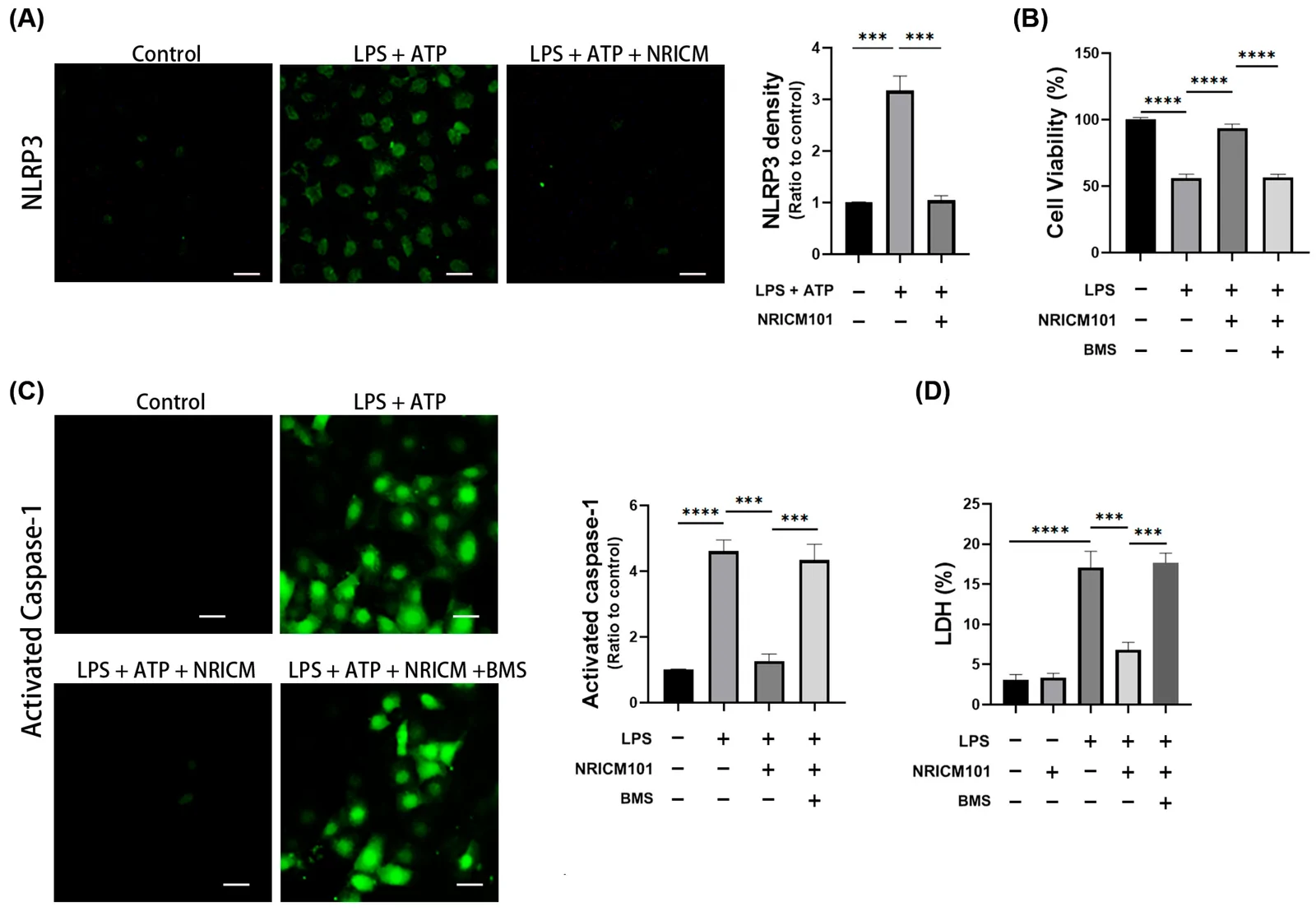
Effects of NRICM101 on NLRP3/caspase-1-associated signaling in NRK52E cells. Cells were treated with LPS (1 μg/mL) and ATP (5 mM) for 24 h, either in the presence or absence of NRICM101 (0.5 mg/mL). For the BMS-986299-treated group, cells were treated with BMS-986299 (10 μM) 1 h before LPS/ATP exposure in the presence of NRICM101 (0.5 mg/mL). (**A**) Representative immunofluorescence staining of NLRP3 expression. (**B**) Cell viability, (**C**) activated caspase-1, and (**D**) LDH release, including an NRICM101-alone group (0.5 mg/mL), were evaluated. Scale bar = 25 μm. (*n* = 4) *** *p* < 0.001, **** *p* < 0.0001.

NLRP3 acts downstream of NF-κB and promotes pyroptosis induced by LPS. After LPS treatment, NLRP3 expression was markedly elevated in the cells (Figure 7A). When cells were treated with NRICM101 during LPS exposure, NLRP3 expression was kept at a baseline level.

To further investigate whether NRICM101 modulates NLRP3/caspase-1-associated signaling and cell injury, cells were treated with NRICM101 and BMS-986299, an NLRP3 agonist. BMS-986299 reversed the protective effects of NRICM101 on cell viability (Figure 7B) and caspase-1 activation (Figure 7C). Consistently, NRICM101 reduced LPS/ATP-induced LDH release, whereas NRICM101 alone did not alter basal LDH release. BMS-986299 reversed the inhibitory effect of NRICM101 on LDH release (Figure 7D), further supporting the involvement of NLRP3-associated signaling.

### 3.8. NRICM101 Attenuates Oxidative Stress and Mitochondrial Dysfunction Associated with NLRP3 Signaling in NRK52E Cells

**Figure 8:**
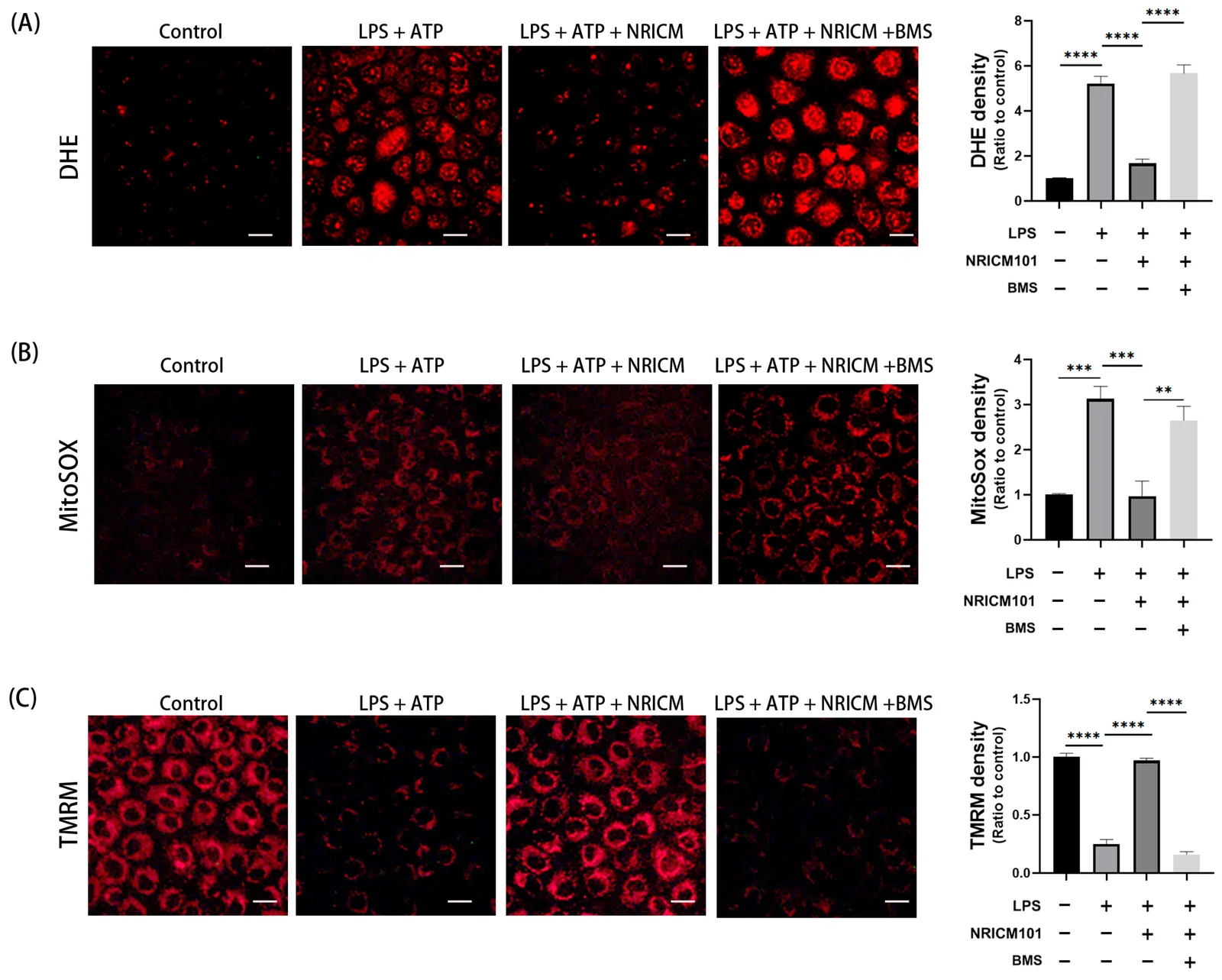
Effects of NRICM101 on oxidative stress and mitochondrial function in NRK52E cells. Cells were treated with LPS (1 μg/mL) and ATP (5 mM) for 24 h, either in the presence or absence of NRICM101 (0.5 mg/mL). For the BMS-986299-treated group, cells were treated with BMS-986299 (10 μM) 1 h before LPS/ATP exposure in the presence of NRICM101 (0.5 mg/mL). (**A**) Intracellular ROS production was assessed by DHE staining. (**B**) Mitochondrial ROS production was assessed using MitoSOX. (**C**) Mitochondrial membrane potential was assessed using TMRM. Scale bar = 25 μm (*n* = 4) ** *p* < 0.01, *** *p* < 0.001, **** *p* < 0.0001.

To determine whether NRICM101 attenuates LPS-induced oxidative stress in NRK52E cells, intracellular ROS (Figure 8A) and mitochondrial ROS (Figure 8B) levels were measured. NRICM101 reduced LPS-induced intracellular and mitochondrial ROS production, whereas the NLRP3 agonist BMS-986299 reversed these effects, suggesting that NLRP3 signaling contributes to ROS generation.

Oxidative stress can also impair mitochondrial function. Mitochondrial health was evaluated by measuring the mitochondrial membrane potential (Figure 8C). NRICM101 preserved mitochondrial membrane potential following LPS exposure, whereas this effect was abolished by BMS-986299, suggesting the involvement of NLRP3-associated signaling in the mitochondrial protective effects of NRICM101.

### 3.9. Identification of the Bioactive Constituents of NRICM101

**Figure 9:**
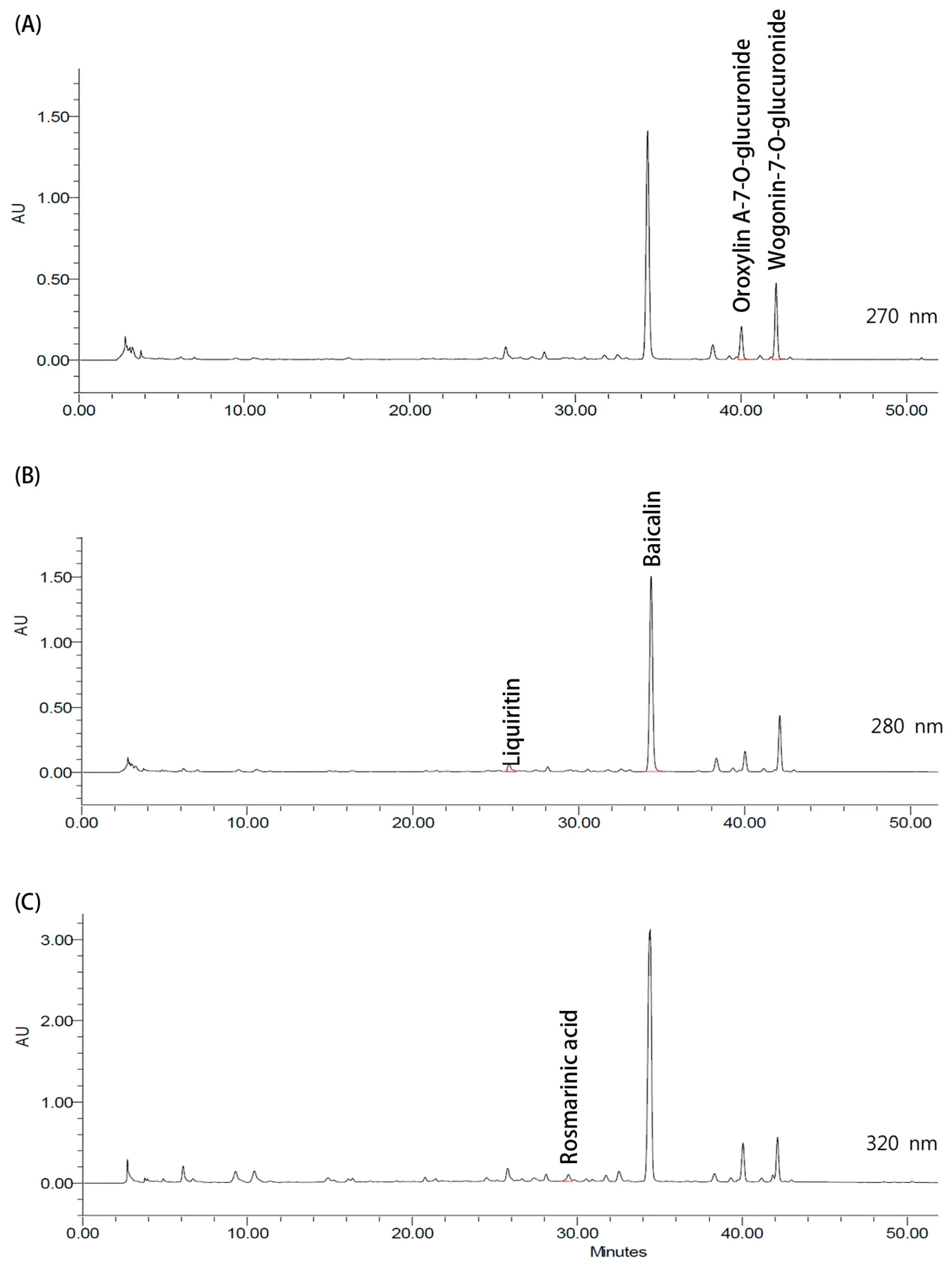
Bioactive compounds in NRICM101 identified using HPLC. The detection wavelengths were (**A**) 270 nm, (**B**) 280 nm, and (**C**) 320 nm.

The chromatographic profile of NRICM101 is shown in Figure 9. The contents of wogonin-7-O-glucuronide, baicalin, liquiritin, oroxylin A-7-O-glucuronide, and rosmarinic acid in the freeze-dried extract of NRICM101 were determined to be 5.09, 27.36, 0.38, 2.83, and 0.57 mg/g, respectively. Among the five quantified constituents, baicalin was the most abundant (27.36 mg/g extract), further supporting its use as a representative chemical marker for characterization of the NRICM101 preparation.

### 3.10. Molecular Docking Analysis of Selected NRICM101 Constituents with Inflammation-Related Targets

**Figure 10:**
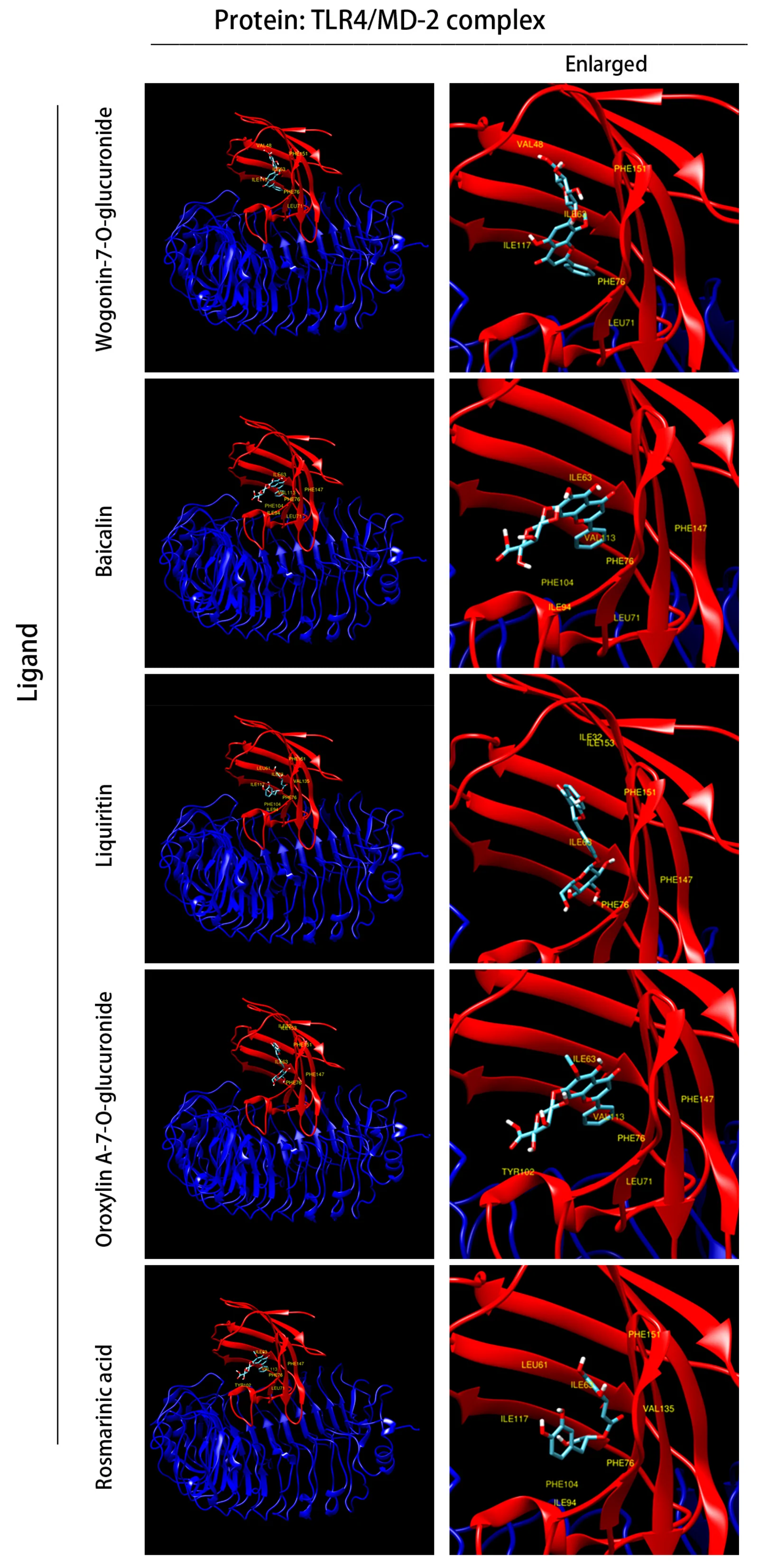
Molecular docking analysis of bioactive constituents of NRICM101 with the TLR4/MD-2 complex (PDB ID: 3FXI). Representative optimal docking poses of the ligands within the MD-2 LPS-binding pocket are shown. MD-2 and TLR4 are shown in red and blue, respectively.

Molecular docking was performed to evaluate the interactions of NRICM101 constituents with the MD-2 LPS-binding pocket of the TLR4/MD-2 complex (PDB ID: 3FXI). Figure 10 presents the optimal binding poses of the identified compounds. Wogonin-7-O-glucuronide, baicalin, liquiritin, oroxylin A-7-O-glucuronide, and rosmarinic acid exhibited predicted docking scores of −8.34, −7.73, −8.08, −7.69, and −7.63 kcal/mol, respectively. These findings suggest potential interactions between NRICM101-derived compounds and the MD-2 LPS-binding pocket.

## 4. Discussion

NRICM101 administered after LPS exposure ameliorated acute renal injury, as evidenced by improved renal function, attenuated histopathological alterations, and reduced inflammatory responses and oxidative stress. These effects were accompanied by reduced p65 phosphorylation and inflammatory mediator expression, as well as decreased NLRP3 and cleaved caspase-1 expression and lower IL-1β and IL-18 mRNA levels. Consistent with the in vivo findings, NRICM101 attenuated NLRP3/caspase-1-associated signaling and reduced LDH release in LPS/ATP-stimulated NRK-52E cells. These findings are consistent with the established role of NF-κB/NLRP3 signaling in LPS-induced renal inflammation and injury [24]. Increased renal NLRP3 expression has also been associated with macrophage infiltration [25], renal fibrosis, and chronic changes following severe AKI [26]. Notably, NRICM101 was administered after an LPS challenge, whereas many experimental studies have evaluated interventions administered before LPS exposure [27,28]. Thus, the present findings demonstrate the protective effects of NRICM101 when administered after the initiation of LPS-induced acute inflammatory renal injury under the experimental conditions used in this study.

NRICM101 markedly attenuated oxidative stress in LPS-induced AKI, as evidenced by reduced MDA and NO levels and preserved SOD, catalase, and GPx activities. In addition, NRICM101 restored the LPS-induced reduction in serum total antioxidant capacity, providing further evidence for its antioxidant effects in vivo. Consistently, in LPS/ATP-stimulated NRK-52E cells, NRICM101 reduced intracellular and mitochondrial ROS accumulation and preserved mitochondrial membrane potential. These findings are consistent with the established interplay between oxidative stress, mitochondrial dysfunction, and NLRP3 activation in AKI [29]. Pharmacological activation of NLRP3 by BMS-986299 attenuated the effects of NRICM101 on ROS accumulation and mitochondrial membrane potential, suggesting that NLRP3-associated signaling may contribute to the antioxidant and mitochondrial-protective effects of NRICM101. This observation is consistent with previous studies showing that NLRP3 activation can exacerbate mitochondrial dysfunction and oxidative stress [30,31] and further supports the reciprocal relationship among these processes during renal injury [32,33,34,35]. NRICM101 is a multi-component herbal formulation containing several phytochemical constituents with intrinsic antioxidant properties, which may directly contribute to the reduction in oxidative stress and cellular injury observed in this study. Although NRICM101 preserved renal antioxidant enzyme activities and increased serum total antioxidant capacity, the present experiments cannot determine the relative contributions of direct antioxidant activity and signaling-mediated regulation of endogenous antioxidant defenses. The cytoprotective and nephroprotective effects of NRICM101 are therefore likely multifactorial and cannot be attributed to a single signaling pathway. These findings suggest that NRICM101 may protect against LPS-induced renal injury through attenuation of oxidative stress, preservation of mitochondrial function, and modulation of inflammation- and NLRP3-associated signaling.

HPLC analysis of NRICM101 identified and quantified five selected constituents, with baicalin being the most abundant. Baicalin and wogonin-7-O-glucuronide, major flavonoid constituents derived from *Scutellaria* Roots, have been reported to exert anti-inflammatory and antioxidant activities [36,37,38], which may partially contribute to the biological effects of NRICM101 observed in the present study. However, the present HPLC analysis was limited to five selected constituents and therefore provides only a partial characterization of the chemical composition of NRICM101. Although the formulation was prepared using fixed herb proportions and controlled extraction conditions, further comprehensive chemical characterization and evaluation of batch-to-batch consistency would strengthen the standardization and quality control of NRICM101 in future studies.

The molecular docking findings provide additional insight into the potential contribution of NRICM101 constituents to its anti-inflammatory effects. The predicted binding of these constituents within the MD-2 LPS-binding pocket suggests their potential interaction with the TLR4/MD-2 complex, which may contribute to the attenuation of inflammatory signaling observed following NRICM101 treatment. However, molecular docking provides computational predictions rather than evidence of direct target engagement or functional inhibition of TLR4/MD-2 signaling. Moreover, the docking analysis was performed using individual constituents, whereas the biological effects observed in this study were produced by the whole NRICM101 formulation. Therefore, the docking results should be interpreted as hypothesis-generating, and further experimental studies, such as surface plasmon resonance or cellular thermal shift assays, are required to validate direct binding and its biological relevance.

Several limitations of the present study should be acknowledged. First, NRICM101-only groups were not included to evaluate the same renal, inflammatory, oxidative-stress, and NLRP3/NF-κB-related parameters assessed in the LPS-treated groups. Therefore, the present study cannot determine whether NRICM101 itself affects these baseline parameters. Nevertheless, the safety of NRICM101 alone has been evaluated in a previous comprehensive toxicological study. A single oral dose of 5 g/kg followed by a 14-day observation period showed no significant acute toxicity. In addition, repeated oral administration of NRICM101 at 1.6, 3.2, and 4.8 g/kg/day for 28 days showed no treatment-related mortality or significant organ toxicity, with renal safety evaluated by serum biochemistry, urinalysis, and histopathological examination [39]. Second, several key outcomes were not validated at the protein or functional level. NGAL and KIM-1 were assessed only at the mRNA level, without corresponding protein measurements in kidney tissue or urine. Similarly, IL-1β and IL-18 were evaluated only at the mRNA level, while their mature protein levels or secretion and GSDMD cleavage were not assessed. Although LDH release was evaluated as a functional indicator of cell injury, the absence of GSDMD cleavage analysis limits definitive conclusions regarding pyroptotic cell death. Therefore, the observed findings support the modulation of pyroptosis-associated signaling rather than providing definitive evidence that pyroptotic cell death was inhibited. Third, NRK-52E cells were selected because they are a well-established renal tubular epithelial cell model that has been widely used to investigate LPS-induced renal inflammation and NLRP3 inflammasome activation [40]. However, the in vivo experiments were conducted in mice, whereas NRK-52E cells are rat-derived; thus, potential cross-species differences should be considered when interpreting and extrapolating between the in vitro and in vivo findings. Future studies using mouse-derived or primary renal tubular epithelial cells are warranted to further validate these findings.

## 5. Conclusions

NRICM101 attenuated LPS-induced acute renal injury by reducing inflammatory responses and oxidative stress and preserving mitochondrial function. These protective effects may involve both direct antioxidant activity and modulation of NF-κB/NLRP3-related signaling. Collectively, these findings support the protective effects of NRICM101 against LPS-induced AKI, although further studies are required to clarify the underlying molecular mechanisms and clinical relevance.

## Data Availability

Results of all analyses are included in this published article. The data presented in this study are available on request.

## Supplementary Materials

The following supporting information can be downloaded at https://www.mdpi.com/article/10.3390/life16091442/s1, Table S1: Primer sequences used for RT-qPCR in mouse kidney tissues; Table S2: Primer sequences used for RT-qPCR in rat NRK-52E cells.

## Abbreviations

AKI	Acute kidney injury
BUN	Blood urea nitrogen
GSDMD	Gasdermin D
GPx	Glutathione peroxidase
H&E	Hematoxylin and eosin
HPLC	High-performance liquid chromatography
IL-1β	Interleukin-1β
IL-18	Interleukin-18
iNOS	Inducible nitric oxide synthase
KIM-1	Kidney injury molecule-1
LPS	Lipopolysaccharide
NRICM101	Taiwan Chingguan Yihau
NF-κB	Nuclear factor kappa-B
NGAL	Neutrophil gelatinase-associated lipocalin
NLRP3	NOD-like receptor protein 3
NO	Nitric oxide
PAS	Periodic acid–Schiff
p-p65	Phosphorylated p65
ROS	Reactive oxygen species
SCr	Serum creatinine
SOD	Superoxide dismutase
TLR4	Toll-like receptor 4
TNF-α	Tumor necrosis factor-α
