# Supplementary material for: Renoprotective Effects of a Herbal Formulation Against Lipopolysaccharide-Induced Acute Kidney Injury Through Anti-Inflammatory and Antioxidant Activities
Source: Life (Basel). 2026 Aug 30;16(9):1442. doi: 10.3390/life16091442 (PMC13608523; doi:10.3390/life16091442)
Supplement: Supplementary file 1 [file life-16-01442-s001.zip › life-4484813-supplementary.pdf]

Table S1. Primer sequences used for RT-qPCR in mouse kidney tissues

Gene	Forward primer (5′–3′)	Reverse primer (5′–3′)	GenBank accession number	Expected amplicon size (bp)
TNF	CGATCACCCCGAAGTTCAGTA	GGTGCCTATGTCTCAGCCTCTT	NM_001278601.1	84
IL-18	CAGCCTGTGTTCGAGGATATG	TCACAGCCAGTCCTCTTACT	NM_008360.2	109
NLRP3	AGAGCCTACAGTTGGGTGAAATG	CCACGCCTACCAGGAAATCTC	NM_145827.4	116
IL-1β	TTCCCCAGGGCATGTTAAGG	GTCTTGGCCGAGGACTAAGG	NM_008361.4	86
NOS2	GGGACTGAGCTGTTAGAGACAC	CCAACGTTCTCCGTTCTCTTG	NM_010927.4	100
Ptgs2	TCCCATGGGTGTGAAGGGAAA	TGGGGGTCAGGGATGAACTC	NM_011198.5	93
Kim1	ACATATCGTGGAATCACAACGAC	ACTGCTCTTCTGATAGGTGACA	NM_001166632.1	114
Ngal	ATGCACAGGTATCCTCAGGT	TGGCGAACTGGTTGTAGTCC	NM_008491.2	70
HPRT	GTTGGGCTTACCTCACTGCT	TAATCACGACGCTGGGACTG	NM_013556.2	125

Table S2. Primer sequences used for RT-qPCR in rat NRK-52E cells

Gene	Forward primer (5′–3′)	Reverse primer (5′–3′)	GenBank accession number	Expected amplicon size (bp)
TNF	ATGGGCTCCCTCTCATCAGT	GCTTGGTGGTTTGCTACGAC	NM_012675.3	106
IL-18	GCCATGTCAGAAGAAGGCTCT	GGATTCGTTGGCTGTTCGGT	NM_019165.2	231
NLRP3	GACCAGCCAGAGTGGAATGATG	CTGTTGAGGTCCACGCTCTC	NM_001191642.1	224
PTGS2	CTCAGCCATGCAGCAAATCC	GGGTGGGCTTCAGCAGTAAT	NM_017232.4	172
NOS2	TGGTGAGGGGACTGGACTTT	TGTTGGGCTGGGAATAGCAC	NM_012611.4	241
IL-1β	CTTTGAAGAAGAGCCCGTCC	CCAAGGCCACAGGGATTTTG	NM_031512.2	242
HPRT	CTTCCTCCTCAGACCGCTTT	TCACTAATCACGACGCTGGG	NM_012583.2	79
